# Expression of Y-box-binding protein YB-1 allows stratification into long- and short-term survivors of head and neck cancer patients

**DOI:** 10.1038/bjc.2011.491

**Published:** 2011-11-17

**Authors:** A Kolk, N Jubitz, K Mengele, K Mantwill, O Bissinger, M Schmitt, M Kremer, P S Holm

**Affiliations:** 1 Department of Oral and Maxillofacial Surgery, Technische Universität München, Klinikum rechts der Isar, Ismaninger Str. 22, 81675, Munich, Germany; 2 Institute of Experimental Oncology, Technische Universität München, Klinikum rechts der Isar, Ismaninger Str. 22, 81675, Munich, Germany; 3 Klinische Forschergruppe, Frauenklinik, Technische Universität München, Klinikum rechts der Isar, Munich, Germany; 4 Institute of Pathology, Technische Universität München, Munich, Germany; 5 XVir Therapeutics GmbH, Munich, Germany

**Keywords:** HNSCC, disease-specific survival, YB-1, prognosis, stratification

## Abstract

**Background:**

Histology-based classifications and clinical parameters of head and neck squamous cell carcinoma (HNSCC) are limited in their clinical capacity to provide information on prognosis and treatment choice of HNSCC. The primary aim of this study was to analyse Y-box-binding protein-1 (YB-1) protein expression in different grading groups of HNSCC patients, and to correlate these findings with the disease-specific survival (DSS).

**Methods:**

We investigated the expression and cellular localisation of the oncogenic transcription/translation factor YB-1 by immunohistochemistry on tissue micro arrays in a total of 365 HNSCC specimens and correlated expression data with clinico-pathological parameters including DSS.

**Results:**

Compared with control tissue from healthy individuals, a significantly (*P*<0.01) increased YB-1 protein expression was observed in high-grade HNSCC patients. By univariate survival data analysis, HNSCC patients with elevated YB-1 protein expression had a significantly (*P*<0.01) decreased DSS. By multivariate Cox regression analysis, high YB-1 expression and nuclear localisation retained its significance as a statistically independent (*P*<0.002) prognostic marker for DSS. Within grade 2 group of HNSCC patients, a subgroup defined by high nuclear and cytoplasmic YB-1 levels (co-expression pattern) in the cells of the tumour invasion front had a significantly poorer 5-year DSS rate of only 38% compared with overall 55% for grade 2 patients. Vice versa, the DSS rate was markedly increased to 74% for grade 2 cancer patients with low YB-1 protein expression at the same localisation.

**Conclusion:**

Our findings point to the fact that YB-1 expression in combination with histological classification in a double stratification strategy is superior to classical grading in the prediction of tumour progression in HNSCC.

Each year, ∼600 000 individuals worldwide are diagnosed with head and neck squamous cell carcinoma (HNSCC) (Sturgis *et al*, 2004; Haddad and Shin, 2008). Despite recent advances in surgery, radiotherapy, and multimodal treatment regiments, including chemotherapy and cetuximab (Bonner *et al*, 2006), there has been only modest improvement in overall survival over the past 20 years (Rogers *et al*, 2009). Disappointedly, overall cure is achieved in <50% of the patients (Tepper *et al*, 2008; Allum *et al*, 2009). Patients with HNSCC with recurrent or metastatic disease have a dismal prognosis, with median survival rates of only 6–10 months (Bozec *et al*, 2008; Rogers *et al*, 2009). Established major prognostic factors are the presence of loco-regional metastasis, vascular or lymphatic invasion, positive surgical margins, and extra capsular spread of tumour cells from involved lymph nodes into soft tissue of the neck (Forastiere *et al*, 2001). Thus, in current clinical practice, histology-based classification and clinical parameters of HNSCC are the most common predictors of patient survival (Hall *et al*, 2009; Mucke *et al*, 2009). However, tumour-node-metastasis (TNM) staging alone is limited in its clinical capacity to provide information on prognosis and optimal treatment choice for HNSCC.

One objective of predictive oncology is to identify biomarkers that would allow the stratification of patients for treatment while avoiding under or overtreatment and preventing unnecessary therapy-related side effects (Chang and Califano, 2008). Major developments including the identification of specific cancer biomarkers, including p53 and the certain involvement of human papilloma virus in oncogenesis (Ginos *et al*, 2004; Chin *et al*, 2005) has increased our understanding of HNSCC over the last several years. Furthermore, proteins in the epithelial growth factor receptor family as well as proteins involved in cellular proliferation, apoptosis, angiogenesis, and immune response posses prognostic value (Chang and Califano, 2008). Recently published data shows that genes involved in the epithelial-mesenchymal transition (EMT) were also predictive for disease recurrence in HNSCC (Chung *et al*, 2006).

The described cancer biomarker Y-box-binding protein-1 (YB-1) is the focus of the present investigation. Evolutionarily YB-1 is one of the most conserved nucleic-acid-binding proteins (Kohno *et al*, 2003). To date, a large number of scientific articles have left little doubt that YB-1, a multifunctional protein, regulates transcription and translation by shuttling between the cytoplasm and the nucleus influencing tumour growth (Schittek *et al*, 2007), chemotherapy resistance (Bargou *et al*, 1997; Kuwano *et al*, 2004), and EMT (Evdokimova *et al*, 2009). A predominant nuclear localisation of YB-1 protein is associated with intrinsic expression of putative oncogenes, such as human epidermal growth factor receptor 2 (HER2), PIK3CA (gene coding for human p110*α* protein), MET (proto-oncogene coding for hepatocyte growth factor receptor), and epidermal-growth-factor receptor (EGFR) (Janz *et al*, 2002; Berquin *et al*, 2005; Sutherland *et al*, 2005; Finkbeiner *et al*, 2009), and the coordination of DNA excisional repair (Das *et al*, 2007). Moreover, several essential signalling pathways, which are activated in HNSCC, including Stat3/NF-*κ* (Lee *et al*, 2008), PI3K/Akt (Sutherland *et al*, 2005; Astanehe *et al*, 2009), and Mek/Erk, are known to target YB-1 (Coles *et al*, 2005). In view of that, YB-1 is supposed to be an important integral part of different signal transduction pathways and due to its role in cancer it is termed an oncogenic transcription/translation factor (Wu *et al*, 2007).

Nuclear YB-1 is also associated with poor prognosis in various other malignancies, including ovarian, lung, synovial cancer, and glioblastoma (Wu *et al*, 2007). In breast cancer YB-1 has been linked to an aggressive cancer phenotype suggesting that YB-1 may help to re-define high-risk breast cancer (Habibi *et al*, 2008). In breast cancer, YB-1 acts as a biomarker for predicting the efficacy of high-dose chemotherapy (Gluz *et al*, 2009). Increases in YB-1 protein expression has also been described for other cancers, such as osteosarcoma, prostate cancer, pancreatic adenocarcinoma, colorectal carcinoma, and medulloblastoma, indicating the clinical impact of YB-1 for the progression of these malignant diseases (Kuwano *et al*, 2003). Elevated cytoplasmic levels of YB-1 have been linked to an increased risk of recurrence in nasopharyngeal carcinoma within a small group of patients treated by radio- or chemoradiotherapy. However, nasopharyngeal carcinoma is distinct to HNSCC in its epidemiology, biology, clinical behaviour, and treatment options, therefore not comparable to our HNSCC collective that includes classical squamous cell carcinoma head and neck tumours. It has been suggested that despite the differences in tumour biology the overexpression of YB-1 in nasopharyngeal cancer may also have clinicopathological significance as a predictive biomarker in HNSCC patients (Tay *et al*, 2009). Though, it remains still unclear if YB-1 expression together with TMN staging can be used to predict survival in HNSCC at the initial time of tumour diagnosis.

## Patients and methods

### Patients and tissue specimens

Formalin-fixed, paraffin-embedded tumour resection tissue samples, including cervical lymph nodes as well as metastases, obtained from 530 patients were initially taken into account. Information related to disease-specific survival (DSS) was available from 365 patients, which were included into the study. Primary tumours sites included oral cavity, oropharynx, and hypopharynx.

All patients were treated between 1991 and 2006 by surgical resection of the primary tumour with minimum resection margins of 5 mm in all directions. All patients received a concomitant unilateral or bilateral selective neck dissection (levels I–III) initially based on tumour location. Fibro-adipose-lymphoid tissue from levels II and III were submitted for histopathological analysis intraoperatively. Where nodal metastasis (N1 or N2a cancer stage) in level II or III were identified, cervical dissection was converted to a modified radical neck dissection (levels I–V) of the affected side (Robbins *et al*, 2008).

No patients received adjuvant chemotherapy and also no neoadjuvant chemo- or radiochemotherapy. Thus, all patients underwent the same treatment protocol. All pT4 tumours and/or a nodal stage pN1, pN2a,b or pN3 (*n*=227, 59.5%) received adjuvant radiation therapy following the same protocol to a total dose of 65–70 Gy. All patients who refused adjuvant radiation therapy were excluded from the study. Also patients with positive resection margins, contralateral nodal metastasis, contra- or ipsilateral positive nodes larger than ⩾3 cm or those with extra-capsular spread (N2c and higher nodal stage), or those with distant metastasis at the time of surgery or predicted survival < 1 month were not included. Tumour size (pT) and lymph node (pN) categories of the tumours were determined according to the current TNM and UICC (International Union against Cancer) classification. Follow-up was standardised for the whole collective (monthly recall in first postoperative year, six times a year in the second year, four times in the third year up to the end of the fifth year, after that once a year) and was monitored with a minimum follow-up time of 3 years, depending on the patients total lifetime.

Written consent for molecular analysis of their tissue for research purposes was obtained from all patients before surgery. Mucosa of healthy controls and tumour-free mucosa of included cancer patients served as reference control tissue for immunostaining of nuclear and cytoplasmic YB-1 expression. The study was approved by the hospitals’ ethics committee at the Klinikum rechts der Isar, Munich, Germany.

### Preparation of tissue microarrays and immunohistochemical staining

For each of the 365 carcinoma patients, paraffin blocks were selected from the archives of the Institute of Pathology at the Technical University of Munich, Germany. After review of all haematoxylin and eosin stained slides, representative samples (1 mm Ø) of tumour tissues were chosen for tissue mirco arrays (TMAs). From each tumour, samples from the centre of the tumour (TC) (invasive tumour cells without any signs of normal peripheral tissue) as well as samples from the invasion front (IF) (invasive cells infiltrating the histologically normal adjacent mucosa) were selected. For construction of the TMA, core needle biopsy specimens were retrieved from the tumour blocks using a manual arrayer (Beecher Instruments, Sun Prairie, WI, USA) and positioned into a recipient paraffin array block. For the purpose of comparison, adjacent unaffected normal mucosal tissues were chosen for TMA in the same fashion. Consecutive 2 *μ*m sections were then cut from the core samples for immunohistochemical staining of YB-1 expression. Immunohistochemical staining was done by using a fluorescein isothiocyanate (FITC)-labelled rabbit antibody directed against a peptide within the C-terminus of YB-1 protein (CDGKETKAADPPAENS amide according to a previous publication (Gluz *et al*, 2009). After deparaffinisation, rehydration, and washing with Tris-buffered saline (TBS; 0.05 mol l^−1^ Trizma-base, 0.15 mol l^−1^ NaCl; pH 7.6), TMA sections were subjected to epitope retrieval. This was done by pressure cooking (4 min, 10 mmol l^−1^ of citrate buffer, pH 6.0), followed by endogenous peroxidase activity blocking, (with 3% H_2_O_2_ for 20 min, room temperature (RT)) in distilled water. After washing in TBS, sections were incubated with the YB-1-directed antibody (2.6 *μ*g ml^−1^, 1 h, RT) in antibody diluent (DakoCytomation, Hamburg, Germany), then followed by another TBS wash, and incubation with 100 μl of undiluted peroxidase-conjugated sheep F(ab)-anti-FITC (TUNEL-POD; Roche, Mannheim, Germany; 30 min, RT). Staining was visualised by chromogenic substrate 3.3′-diaminobenzidine addition (DakoCytomation; 10 min, RT). Finally, nuclei were then counterstained with Mayer's acid haematoxylin and slides were then covered using Pertex mounting medium (MEDITE, Burgdorf, Germany). As a negative control, this procedure was run omitting the primary antibody. YB-1 immunoreactivity in tumour cells was scored independently by two of the authors (NJ and MK) blinded to the clinical staging of the patients tumours. Photographs of the stained sections were taken with Zeiss Axiophot Microscope (Carl Zeiss AG, Jena, Germany) and edited with Axio Vision Release 4.8 software (Carl Zeiss AG).

### Western blot analysis

Snap-frozen tissue was homogenised according to standard protocol. Protein concentrations were determined using the BCA Protein Assay kit (Pierce, Rockford, IL, USA). Protein (30 *μ*g) was separated on 10% SDS-polyacrylamide gels and electroblotted onto Hybond-ECL nitrocellulose membrane (GE Healthcare, Munich, Germany). The membrane was incubated overnight at 4 °C in TBST (50 mmol l^−1^ Tris-HCl (pH 7.6), 150 mmol l^−1^ NaCl, 0.2% Tween 20) containing 5% fat-free dry milk (AppliChem, Darmstadt, Germany), and then probed with the YB-1 specific antibody (Holzmuller *et al*, 2011) and a YB-1 specific antibody recognising phosphorylated YB-1 (Cell Signaling, Danvers, MA, USA). To demonstrate equal loading an antibody to *α*-tubulin (Calbiochem, San Diego, CA, USA) was used. The bands were visualised using an ECL-based immunochemistry system (Roche, Penzberg, Germany). FaDu (American Type Culture Collection: HTB 43) was kindly provided by Dr O Gires (LMU Munich, Germany). CAL 33 (ACC 447) was kindly provided by Dr R Grenman (Turku University Hospital, Turku, Finland). CAL 33 and FaDu are derived from moderately differentiated squamous cell carcinomas from the tongue and hypopharynx, respectively.

### Scoring of YB-1-related immunoreactivity

Semi-quantitative scoring for YB-1 immunoreactivity was carried out as described previously (Janz *et al*, 2002). This scoring method assesses the percentage of malignant cells that show cytoplasmic and nucleic staining within a tissue core. YB-1 staining of normal cells in adjacent benign mucosa tissue areas was defined as background and subtracted from the scores within the tumour tissue, resulting in value that reflects ‘overexpression’ within tumour areas for further analysis. Scoring results of the tumour areas were classified as follows: score 0 – no tumour cells with immunoreactivity; score +1 – weak reactivity, <20% of tumour cells positive; score 2+ – moderate activity, 20–50% of tumour cells positive; score 3+ – strong activity with 50–80% of tumour cells positive; score 4+ – strong to complete activity, 80–100% of tumour cells positive.

### Statistical analyses

Protein expression values for YB-1 were correlated with pT, pN status, histological grading (G) and DSS. Descriptive statistics for quantitative variables are given as the mean±s.d. and where appropriate, as medians and ranges.

Tumour site, pT, and nodal status as well as the addition adjuvant radiation therapy were randomised within the subgroups to exclude radiotherapy as a confounder.

Death was categorised as death due to HNSCC. For this analysis, the 5-year DSS in months was used as a dependent variable. For DSS analysis, patients were followed clinically from the time of initial diagnosis until their last tumour-free clinical follow-up appointment. DSS was compared with high and low nuclear *vs* cytoplasmic YB-1 expression at the IF and TC using Kaplan–Meier estimates and the log-rank test for equality of survival curves. The correlation coefficient of TNM classification, tumour grade, and YB-1 expression was calculated using the Spearman's rank test. Independent prognostic relevance of high YB-1 immunoreactivity and the association with probabilities of disease recurrence and DSS were adjusted using Cox proportional hazards regression models together with age, gender, histopathological tumour grade, and TNM/UICC classification. A bivariate analysis of YB-1 expression was performed where ‘high’ (score 3 or 4) *vs* ‘low’ (scores 0 to 2) were used. All *P*-values given are unadjusted, two-sided, and subject to a significance level of 5%. All data were analysed and figures (including plots of the Kaplan-Meier survival curves) were generated by ‘Statistical Package for the Social Sciences’ (SPSS for Windows, release 18.0.0, 2009, SPSS Inc., Chicago, IL, USA).

## Results

### Clinical characteristics of the HNSCC patients

DSS data was available for 365 HSNCC patients. The majority of the patients were >50 years of age at time of primary surgery (*n*=352, 96.4%) with a male predominance (*n*=291, 79.7%).

Of the latter 281 (77%) patients were tumour stage pT1/2. Of those with pT1/2 tumours 148 (40.5%) were node negative. With respect to histologic grading, ∼half of the patients (*n*=202, 55.3%) were classified as having tumours with intermediate histological grade 2 (G2), 136 (37.3%) were high-grade (G3) and 27 (7.4%) had low-grade (G1) tumours (Table 1). None of the patient's tumours were classified as a grade four. Median survival time for G1 was 125 month±28, for G2 73 month±9, and for G3 tumours 62 months±16. Furthermore, the 5-year DSS rate decreased significantly with increasing tumour grade; from 83% in G1 tumours, to 55% in G2 tumours, to 51% in G3 tumours (*P*<0.01). Of the total 365 patients 62 patients (17%) recurred locally within the 5-year follow-up period. In all, 45 (72.6%) of those 62 patients developed their recurrence within the first 24 months after surgery (*P*<0.001). The median number of months to recurrence for all of 365 patients was 31.4 within the follow-up period. Death occurred due to local or cervical tumour recurrence, distant metastasis, or secondary oral cavity carcinoma in 192 of 365 patients. Distant metastases were identified in 64 of 365 derived from the primary or a recurrent HNSCC during the total study time of 156 months.

### Protein expression analysis of YB-1 in HNSCC tumour tissue specimens

**Figure 1:**
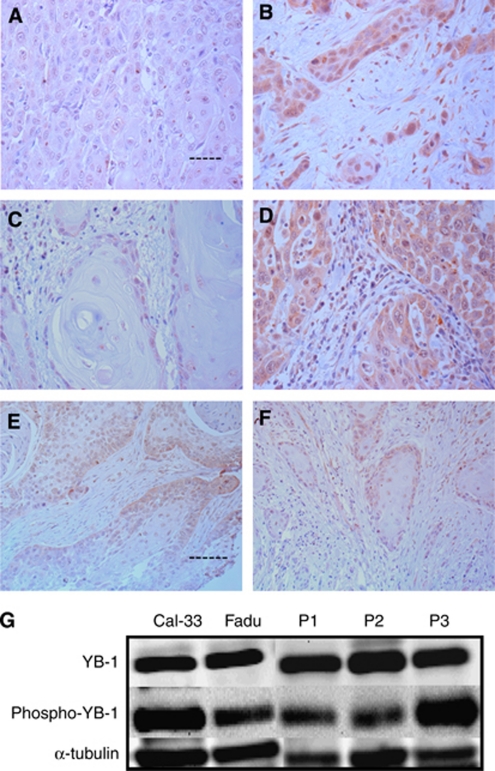
Immunoreactivity of YB-1 protein in healthy control tissue (**A**) and poorly differentiated HNSCC in TC and IF (**B**, **C** and **D**: scale bar 0.05 mm; **E** and **F**: scale bar 0.06 mm). Absent to minimal expression of cells (no score) (**A**). IF: strong nuclear expression of the tumour cells (score 3+) (**B**). Example of a well-differentiated HNSCC, with a faint to moderate staining in the tumour cells nearby the unaffected connective tissue (score 1+) (**C**). TC: strong nuclear and cytoplasmic expression of tumour cells (score 3+) (**D**). IF: weak nuclear and cytoplasmic expression of tumour cells (score 1+) (**E**). IF: moderate nuclear and cytoplasmic expression of tumour cells (score 2+) (**F**). Western blot showing YB-1 protein and phosphorylated YB-1 expression in different tumour tissues (P1-P3) and human HNSCC cell lines FaDu and CAL-33. α-tubulin served as a loading control (**G**).

Results of the investigation of YB-1 expression in the 365 patients with HNSCC from the centre of the tumour, the IF, unaffected oral mucosa, and the oral mucosa of 10 healthy individuals were as follows. Strong staining for YB-1 protein was observed in cancer cells of the oral cavity of most of the HNSCC patients. In the cases representing the TC YB-1 staining was located in the nucleus of tumour tissue cells 88% of the time and cytoplasmic staining was observed in 63% of the cases. When looking at the tumour samples from the IF YB-1 protein expression was located in the nucleus 90% of the time and less in the respective cytoplasm (67%). Nuclear and cytoplasmic YB-1 protein expression in tumour cells of the HNSCC tissue samples was a feature of over 60% of the HNSCC cases (in the TC and IF). In contrast, faint or no YB-1 expression was observed in normal intraoral mucosa of healthy controls as well as in unaffected tumour-free mucosa of the investigated patients. (Figure 1) (A-F) indicates representative immuno-histochemical staining of variant YB-1 expression intensities in HNSCC.

**Figure 2:**
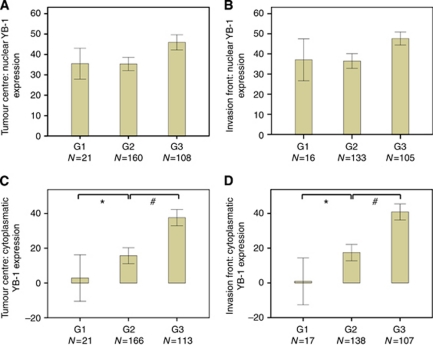
Nuclear (**A** and **B**) and cytoplasmic (**C and D**) YB-1 protein expression in HNSCC tumour tissue cells in the TC (**A** and **C**) and at the IF (**B** and **D**) in relation to histological grading G1 to G3 (^*^P<0.05: G2 to G1; ^#^*P*<0.01: G3 to G2).

**Figure 3:**
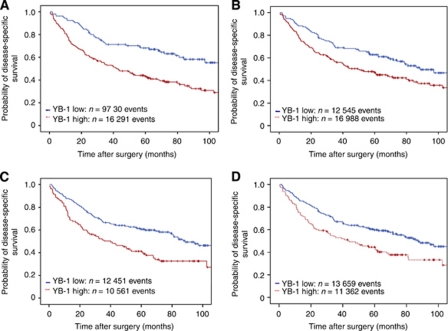
Kaplan–Meier plots illustrating the impact of YB-1 expression on DSS in the HNSSC cohort recalled for more than 60 months after surgery. Low YB-1 expression: upper blue line. High YB-1 expression: lower red line. Median DSS in patients with high/low nuclear YB-1 expression at the IF (**A**). Median DSS in patients with high/low nuclear YB-1 expression in TC (**B**). Median DSS in patients with high/low cytoplasmic YB-1 expression at the IF (**C**). Median DSS in patients with high/low cytoplasmic YB-1 expression in TC (**D**). The colour reproduction of this figure is available at the *British Journal of Cancer* online.

In parallel, for several YB-1-positive cases selected at random, the protein was extracted from fresh-frozen tumour tissue samples and subjected to western blot analysis (Figure 1G). All three patients had tumour tissue extracts showing a YB-1 staining pattern similar to that of the oral cancer reference cell lines FaDu and CAL-33. Phosphorylated YB-1 could also be detected in all cases, indicating the presence of nuclear YB-1 in the tumour tissue of these selected patients. With respect to tumour grade and YB-1 expression, a linear relationship was not observed. Figure 2 depicts the lack of statistical difference between G1, G2, and G3 tumours for YB-1 nuclear protein expression, whether expressed in the TC (Figure 2A) or in the IF (Figure 2B). However, a statistically significant increase in cytoplasmic YB-1 protein expression is demonstrated in increasing order from G1 to G3 (Figures 2C and 3D).

### Protein expression of YB-1 in relation to DSS

Based on the expression pattern of YB-1 we found that elevated nuclear YB-1 expression was associated with decreased survival of the HNSCC patients, with statistical difference (*P*=0.008 for TC samples; *P*<0.001 for tumour IF samples) (Figures 3A and B). A low DSS rate of 41% was observed with the presence of high nuclear YB-1 expression at the IF in comparison to 67% DSS with low YB-1 expression (Figure 3A). To further verify the impact of YB-1 expression on survival, the cytoplasmic localisation of YB-1 was investigated as well. Corresponding to the data derived from nuclear expression, increased cytoplasmic YB-1 levels were also found to have a significant impact on the 5-year DSS rate (*P*=0.005 for TC; *P*=0.001 for IF) (Figures 3C and 3D).

### Correlation of YB-1 expression to DSS based on histologic grading

YB-1 expression and localisation within the different grading categories was analysed. Elevated nuclear expression of YB-1 independent of its location within the tumour was associated with significantly poorer survival of the HNSCC patients. In patients with G1 tumours the 5-year DSS rate was 89 and 85% when nuclear YB-1 expression was low at the IF and the TC, respectively, *vs* a 71% 5-year DSS for G1 grade tumours when YB-1 expression was high. For grade 2 tumours the differences were statistically significant, independent of tumour location, or the site of YB-1 expression. For G2 grade tumours the 5-year DSS for low YB-1 expression was 67% and 60% for centre and IF, respectively, compared with 39 and 44% in the high expression group. In the G3 group 5-year DSS for low YB-1 expression were 61 and 56% for TC *vs* IF compared with 43% (46%), respectively, in the high YB-1 expression group (Table 2). Results obtained for cytoplasmic YB-1 expression for TC also showed a tendency to shorter DSS but with less clarity. These results indicate that YB-1 expression at the IF is a more sensitive parameter for DSS than YB-1 expression in the TC.

### Subgroup analysis of G2 tumours with respect to long-term and short-term DSS

**Figure 4:**
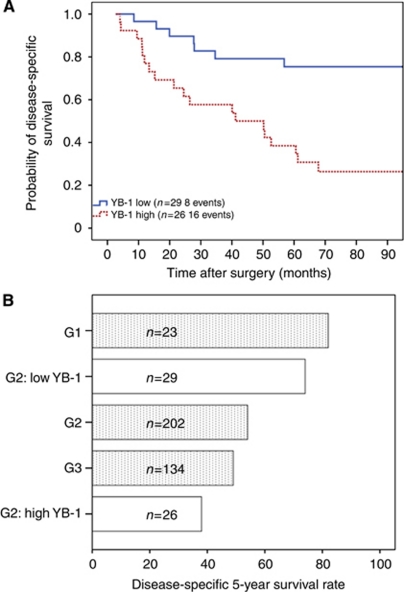
Kaplan–Meier plot illustrating DSS within the subgroup of patients with grade 2 HNSSC tumours sub divided according their YB-1 expression at the IF. Low nuclear and cytoplamatic YB-1 expression: continuous upper blue line; high nuclear and cytoplasmic YB-1 expression: dotted lower red line (**A**). YB-1 protein expression determined by immunohistochemistry identifies low- and high-risk grade 2 HNSCC patients. The 5-year DSS rates of histological grade G1, G2, and G3 patients in comparison to sub divided grade 2 HNSCC patients with low YB-1 expression in the nucleus and cytoplasm, and high nuclear and cytoplasmic YB-1 expression (co-expression pattern) (**B**).

Our initial data show that both nuclear and cytoplasmic YB-1 expression determined correlated with a lower 5-year DSS in HNSCC, with greatest significance at the IF (Figure 3A and C). How the combined nuclear and cytoplasmic staining (co-expression pattern) would correlate with DSS was thus investigated as a subgroup analysis. Due to statistical requirements only the important subgroup G2, which represents the vast majority of the whole collective (and is the most commonly diagnosed histologic category) was therefore further analysed. A statistically significant difference (*P*=0.001) was observed when DSS was plotted against high combined nuclear and cytoplasmic YB-1 expression at the IF *vs* low combined YB-1 protein expression of tumour cells at the IF (Figure 4A). Our results indicate that, grade 2 HNSCC patients (Figure 4B) with low YB-1 protein expression levels have a 5-year DSS rate (74%, *n*=29) similar to the G1 patient subgroup (83%, *n*=23) without YB-1 stratification. The 5-year DSS rate associated with high expression YB-1 G2 patients (38%, *n*=26) is lower than that of the survival G3 subgroup (51%, *n*=134). In further analysis of the low and high YB-1 expression groups within the G2 differentiation 21 patients out of 29 survived 5 years with combined low YB-1 expression. In contrast, only 10 patients out of 26 with high combined YB-1 expression in both compartments, nuclear as well as cytoplasmic, survived. This demonstrates that 51% more patients survived within 5 years when YB-1-combined expression was low (Figure 4A). The survival outcome difference is maintained at 7 years. DSS was as recorded at 70% in the low YB-1 combined expression group and 24% in the high YB-1 combined expression group (data not shown).

### YB-1 protein expression as a statistically independent cancer biomarker in prediction of DSS of HSNCC patients

To evaluate the influence of nuclear YB-1 expression at the IF on DSS and established clinical and histomorphological parameters a multivariate Cox regression analysis was performed (Table 3). In addition to lymph node status (hazard ratio (HR)=1.45), elevated nuclear YB-1 expression at the IF was the most significant variable predicting DSS (HR=2.172). Age, histopathologic grade, and pT were not found to be statistically significant predictors of survival. A log-rank-test was also used as a second statistical model. This evaluation showed that tumour size pT (*P*=0.022) and nodal stage pN (*P*=0.001) also had a significant correlation with the DSS. Furthermore, for patients with high combined nuclear and cytoplasmic YB-1 expression at the IF (co-expression pattern group), the risk of lower DSS even further increases (HR=2.747) (Table 3).

We also analysed the correlation between YB-1 protein expression in tumour cells located at the tumour IF and those demonstrating co-expression in different subgroups defined by histological grade, pT, and nodal status at 5-year follow-up. The *χ*^2^-test showed that pT and pN, significantly correlate positively with cytoplasmic and nuclear YB-1 expression at the TC and at the tumour IF, respectively, within the entire cohort (*P*<0.001). The log-rank test demonstrated that for T1 and T2 patients the 5-year DSS rate is influenced significantly by YB-1 protein expression in the nucleus or co-expression pattern of the nucleus plus cytoplasm. Increased levels of YB-1 protein are associated with significantly lower DSS, for pT1 (*P*=0.014) and pT2 (*P*=0.001) tumours (Table 4). For all node-negative HNSCC patients low YB-1 protein expression is associated with higher 5-year DSS rate, which is statistically significant for nuclear YB-1 protein expression at the IF (N0, *P*=0.001) and supported for the nuclear plus cytoplasmic co-expression of YB-1 demonstrating a trend without statistical significance (Table 4).

## Discussion

The current knowledge about the molecular mechanisms underlying disease progression and drug resistance in HNSCC is still limited. In addition, conflicting published results and disappointing clinical studies have made single cancer biomarkers highly controversial (Shah *et al*, 2009). A comparative analysis of different markers and clinico-pathological features concerning their prognostic significance in oesophageal adenocarcinoma revealed that only tumour stage and lymph node involvement correlate with the survival of the patient (Langer *et al*, 2006). The grade of cellular differentiation is a rather time-independent statement about the aggressiveness of the tumour (in contrast to DSS). However, it is beyond question that the grade of cellular differentiation of HNSCC to some extent influences survival. Cancer biomarkers that more accurately predict clinical outcome and response to anticancer therapy, in alignment with the established TNM and UICC classification, would still be of great advantage in the management of HNSCC patients (Hundsdorfer *et al*, 2005; Ludwig and Weinstein, 2005).

Similar to the heat-shock proteins, the cold-shock domain protein YB-1 is thought to have a fundamental role in a wide variety of environmental stress reactions (Kohno *et al*, 2003). Increased expression of YB-1 in many solid tumours, such as breast cancer or melanoma, was found to be associated with malignant growth, drug resistance and poor clinical outcome (Bargou *et al*, 1997; Schittek *et al*, 2007). However, the protein expression of YB-1 in reference to the grading and DSS in HNSCC has not been examined in detail. In the present report we present clinically relevant data regarding the clinical impact of YB-1 protein expression in patients by analysing a large representative collective of untreated human tissues obtained from HNSCC patients at primary surgery, which were subjected to YB-1 specific immunohistochemistry. The statistical power associated with such a large sample size of 365 HNSCC patients combined with a follow-up period exceeding 60 months for most of the patients was a major strength of this study.

Protein expression of YB-1 determined by immunohistochemistry in the HNSCC TMAs was significantly increased in the cytoplasm as well as in the nucleus at the IF and in the centre of the tumour of the entire cohort in comparison to normal head and neck mucosal tissue (Figure 1). Interestingly, cytoplasmic but not nuclear YB-1 protein expression correlated with tumour grade in HNSCC specimens, with the highest level of expression in G3 tumours (Figure 2). Comparable studies on the same subject have not been reported so far. The only previous study concerning YB-1 protein expression in a sub-group of HNSCC was conducted in nasopharyngeal carcinoma, which dealt exclusively with cytoplasmic YB-1 protein expression (Tay *et al*, 2009). Other studies conducted in the breast, ovarian cancer, and non-small cell lung cancer tissue specimens as well as osteosarcoma have in a manner similar to our data confirmed the presence of concomitant nuclear YB-1 expression together with cytoplasmic localisation of YB-1 (Bargou *et al*, 1997; Oda *et al*, 1998, 2003; Kamura *et al*, 1999; Shibahara *et al*, 2001).

In the next step we analysed the involvement of YB-1 expression on the 5-year DSS rates. Univariate data analyses of patients with high YB-1 expression, regardless of their within-cell localisation, were afflicted with significantly decreased 5-year DSS rates. This statistically significant result implicating a strong correlation between YB-1 expression and prognosis of the HNSCC patients may have valuable future clinical importance (Figure 3). From the multivariate analysis, which included established clinical and histomorphological factors and YB-1 staining status, translocation of YB-1 protein into the nucleus of tumour cells at the IF emerged as a statistically independent prognostic biomarker, in addition to lymph node involvement. In this context, age, histological grading, and pT were of no statistical relevance (Table 3A). Taken together, our data show clearly that nuclear YB-1 affects the DSS rates in HNSCC and is an independent prognostic biomarker. However, the results also open the interesting question why cytoplasmic YB-1 but not nuclear YB-1 correlates with grade of HNSCC.

The observed impact of nuclear YB-1 on the clinical outcome is in line with data found in prostate cancer, non-small-cell lung carcinoma and B-cell lymphoma showing that YB-1 also is a cancer biomarker for tumour aggressiveness (Shibahara *et al*, 2001; Gimenez-Bonafe *et al*, 2004; Habibi *et al*, 2008; Xu *et al*, 2009). Additionally, YB-1 expression in breast cancer has been studied intensively, where strong clinical evidence suggests that YB-1 protein expression and its translocation from the cytoplasm to the nucleus is linked with tumour progression and could be used as an independent biomarker for aggressive breast cancer across all subtypes (Habibi *et al*, 2008).

Based on these results, we explored whether YB-1 protein expression could be used as a cancer biomarker for a more accurate sub-classification of high-risk HNSCC cancer patients. For this, different grading groups were separately analysed with regard to DSS and YB-1 protein expression. As shown in Table 2, in particular, the vast majority of the cohort of patients with G2 tumours with high YB-1 expression at the tumour IF, exhibited a statistically significant, shorter, DSS compared with patients with low YB-1 protein expression. This suggests that YB-1 protein expression in tumour cells located at the IF of the tumour in combination with histological grading improves prediction of clinical outcome. This might influence selection of patients who will benefit from adjuvant chemo/radiotherapy in a cohort of patients for whom it may not traditionally have been indicated. Our results are in line with data obtained by (Bryne *et al*, 1992), showing that the invasive front grading system is of high prognostic value for HNSCC. It should be noted, that nuclear and cytoplasmic YB-1 expression in the high and low grading groups G3 *vs* G1 has not shown statistically significant correlation with DSS. This may be due to low number of patients in G1 histologic subgroup and inhomogeneous nature of the G3 subgroup. However, despite a lack of statistical significance a tendency was observed.

In contrast to other YB-1 biomarker studies (To *et al*, 2010), which consider nuclear YB-1 only, we also looked at the cytoplasmic expression in the evaluation of the DSS of grade 2 HNSCC patients. The application of the co-expression pattern of YB-1 at the IF in the survival analyses further amplified the discrimination of patients with grade 2 HNSCC tumours between ‘short’- and ‘long’-term survivors (Figure 4B). More specifically, the 5-year DSS record from both patient groups revealed that nearly 50% of the patients being considered as medium risk based on their histologic tumour grade were more likely to die of the disease than expected if they demonstrated high YB-1 combined expression. The data suggest that YB-1 protein expression assessed by immunohistochemistry leads to an ameliorated identification of high-risk patients. This may lead to more individualised therapy of HNSCC cancer and should be included in the post surgical analysis of grade 2 HNSCC patients to allow individualised treatment decisions with respect to adjuvant therapy in this important patient subgroup. We would suggest that before entering clinical practice, however, validation of the data in another set of HNSCC patients is essential.

It is established that YB-1 expression and localisation could affect many critical aspects of tumour biology as it is involved in fundamental processes such as mRNA splicing (Stickeler *et al*, 2001), cell cycle (Jurchott *et al*, 2003), drug resistance (Bargou *et al*, 1997), DNA repair (Gaudreault *et al*, 2004) and translational regulation (Evdokimova *et al*, 2001). Upon nuclear translocation, which is believed to be mediated by Akt and Erk through phosphorylation (Coles *et al*, 2005; Sutherland *et al*, 2005), YB-1 regulates the transcription activity by binding to gene promoter regions containing the Y-box motif, and among others, YB-1 thus may activate gene expression of the EGFR (Stratford *et al*, 2007), matrix metalloproteinase 2 (Mertens *et al*, 1997), and MET (Finkbeiner *et al*, 2009), which are associated with tumour cell adhesion, invasion and metastasis in HNSCC.

The most important two recent observations include the following: (i) cytoplasmic YB-1 involvement in EMT (Evdokimova *et al*, 2009), which has a key role in cancer invasion and metastasis and (ii) YB-1 induction of CD44 expression (To *et al*, 2010), which is a widely recognised marker for initiation of cancer cell development including initiation of the HNSCC cancer process (Prince *et al*, 2007). It has been suggested that inhibition of YB-1 expression could thus minimise cancer recurrence (To *et al*, 2010).

YB-1 expression is regulated by Twist (Shiota *et al*, 2009) and interferes with the mTor/Stat3 pathway (Lee *et al*, 2008). Both pathways possess prognostic relevance (Shah *et al*, 2006; Xie *et al*, 2009; Masuda *et al*, 2010) and are known to have an important role in EMT, the maintenance of cancer initiating cells, and multidrug resistance (Yang *et al*, 2004; Li *et al*, 2009; Sherry *et al*, 2009). In addition, results obtained by Chung *et al* (2006) show that the molecular determinants of EMT and NF-*κ*B are characteristics of high-risk HNSCC tumours . NF-*κ*B is also involved in regulating Twist expression (Pham *et al*, 2007) and enhancing Stat3 activity in HNSCC (Masuda *et al*, 2010). Thus, besides YB-1, proteins such as Stat3 and Twist, hypoxia-inducible factor-*α*, and Slug have also been identified as markers of malignant progression (Ginos *et al*, 2004; Jethwa *et al*, 2008; Huang *et al*, 2009) and may be candidates for cancer biomarkers to be looked at in a future HNSCC biomarker screening programme.

In the clinical setting, in addition to prediction of therapy response, adequate risk-group assessment is a prerequisite for an individualised therapy concept for HNSCC (Conley, 2006). This report shows that determination of YB-1 protein expression and its intracellular localisation in tumour cells at the IF are valuable molecular tools to classify grade 2 HNSCC patients into long- and short-term survivors. This may allow offering of more optimal therapeutic options for this subgroup of HNSCC patients (Gluz *et al*, 2009). As chemo-radiotherapy is associated with severe toxicities this decision-making process is an important one (Conley, 2006).

In summary, based on our results from a large, homogenous and representative human HNSCC tissue collection, we propose that YB-1 immunohistochemistry analysis should be considered as an integral of the histomorphological diagnostic system. When this is done in conjunction with the classical histological grading system it could allow personalised treatment of high-risk groups of HNSCC patients.

Taken into account that YB-1 expression is linked to multi drug resistance in various tumour entities so far as well as against EGFR-tailored drugs (Kashihara *et al*, 2009) YB-1 expression was also found to be also involved in cancer stem cell biology (To *et al*, 2010) and trastuzumab resistance (Dhillon *et al*, 2010). In light of these findings and our recent observations that YB-1-based virotherapy kills cancer stem cells equipped with nuclear YB-1 expression (Mantwill *et al*, 2011; manuscript submitted to Stem Cells), we suggest that YB-1 analysis in conjunction with YB-1 based virotherapy represents an attractive therapeutic strategy for a multimodal treatment concept for high-risk subgroup of HNSCC patients.

## Figures and Tables

**Table 1:** Baseline and treatment characteristics of HNSCC patients and associated histomorphological data

	**Number of cases (%)**
Patients	365 (100)
	
*Age (years)*	
⩽50	13 (3.6)
>50	352 (96.4)
	
*Gender*	
Male	291 (79.7)
Female	74 (20.3)
	
*Tumour size*	
pT1	166 (45.5)
pT2	115 (31.5)
pT3	39 (10.7)
pT4	45 (12.3)
	
*Nodal status*	
pN0	148 (40.5)
pN1	64 (17.5)
pN2	100 (24.4)
pN3	2 (0.6)
pNX	51 (14.0)
	
*Distant metastasis (at any time)*	
Absent	303 (83)
Present	64 (17)
	
*Degree of differentiation (grade)*	
G1	27 (7.4)
G2	202 (55.3)
G3	136 (37.3)
	
*Recurrence at 5 years*	
0	303 (79.7)
+	62 (20.3)
	
5-year DSS	192 (53.0)

Abbreviations: DSS=disease-specific survival; HNSCC=head and neck squamous cell carcinoma.

The term distant metastasis refers to the presentation with any metastasis from the primary or recurrent HNSCC during the whole study duration of 156 months.

**Table 2:** Association of high/low cytoplasmic (A) and nuclear (B) YB-1 expression at the IF or TC on 5-year DSS (%) in relation to histological grading

	**IF**			**TC**		
	**High YB-1**	**Low YB-1**	***P***	**High YB-1**	**Low YB-1**	***P***
*A*						
G1	100	81	*NS*	100	77	*NS*
G2	42	57	*0.031*	38	58	*0.012*
G3	39	62	*NS*	45	53	*NS*
						
*B*						
G1	71	89	*NS*	71	85	*NS*
G2	39	67	*0.001*	44	60	*0.019*
G3	43	61	*NS*	46	56	*NS*

Abbreviations: DSS=disease-specific survival; IF=invasion front; TC=tumour center; YB-1=Y-box-binding protein-1. *P*-values below 0.05 are in italics.

**Table 3:** Multivariate Cox proportional hazards regression analysis to define the statistically independent impact of nuclear YB-1 expression on DSS of HNSCC patients

	***β***	***P***	**Exp (*β*)**	**Lower CI**	**Higher CI**
*A*					
Age	0.016	*NS*	1.016	0.998	1.035
Grading	−0.016	*NS*	0.984	0.694	1.396
Tumour size (T1–T4)	−0.032	*NS*	0.968	0.808	1.161
Lymph node involvement (N0–N2)	0.374	*0.001*	1.454	1.162	1.819
Nuclear YB-1 overexpression IF (low/high)	0.776	*0.002*	2.172	1.343	3.512
					
*B*					
Age	0.030	*0.010*	1.030	1.007	1.053
Grading	0.123	*NS*	1.131	0.662	1.933
Tumour size (T1–T4)	−0.150	*NS*	0.861	0.643	1.152
Lymph node involvement (N0–N2)	0.385	*0.001*	1.469	1.171	1.844
Nuclear and cytoplasmic YB-1 overexpression IF (low/high)	1.011	*0.014*	2.747	1.226	6.155

Abbreviations: CI=confidence interval; YB-1=Y-box-binding protein-1.

Nuclear YB-1 expression at the IF (A) and the co-expression pattern (nuclear and cytoplasmic YB-1 expression) of YB-1 at the IF (B) emerged as a statistically independent cancer biomarker to predict DSS of HNSCC patients. *P*-values below 0.05 are in italics.

**Table 4:** Influence of T and N on 5-year disease-specific survival rate in % (5-year %) depending on solely nuclear or combined nuclear and cytoplasmic YB-1 expression at the IF

	**IF: nuclear YB-1 expression**				**IF: nuclear+cytoplasic YB-1 expression**		
	**YB-1**	**5-year %**	***n***	***P***	**5-year %**	***n***	***P***
*T*							
T1	Low	71	52	*0.014*	74	20	*< 0.001*
	High	50	41		30	23	
T2	Low	70	34	*< 0.001*	80	15	*0.011*
	High	38	56		44	34	
T3	Low	56	5	*NS*	—	2	*NS*
	High	44	20		45	17	
T4	Low	85	7	*NS*	—		—
	High	48	28		55	11	
							
*N*							
N0	Low	86	52	*0.001*	83	24	*NS*
	High	58	44		68	23	
N1	Low	75	17	*NS*	100	2	*NS*
	High	56	29		58	17	
N2	Low	51	19	*NS*	58	10	*NS*
	High	25	54		24	39	

Abbreviations: IF=invasion front; *n*=number of patients at the beginning of 5-year follow-up period; N=nodal status; NS=no statistically significant difference; T=tumour size. *P*-values below 0.05 are in italics.

## References

[bib1] Allum WH, Stenning SP, Bancewicz J, Clark PI, Langley RE (2009) Long-term results of a randomized trial of surgery with or without preoperative chemotherapy in esophageal cancer. J Clin Oncol 27(30): 5062–5067 19770374 10.1200/JCO.2009.22.2083

[bib2] Astanehe A, Finkbeiner MR, Hojabrpour P, To K, Fotovati A, Shadeo A, Stratford AL, Lam WL, Berquin IM, Duronio V, Dunn SE (2009) The transcriptional induction of PIK3CA in tumor cells is dependent on the oncoprotein Y-box binding protein-1. Oncogene 28(25): 2406–2418 19430491 10.1038/onc.2009.81

[bib3] Bargou RC, Jurchott K, Wagener C, Bergmann S, Metzner S, Bommert K, Mapara MY, Winzer KJ, Dietel M, Dorken B, Royer HD (1997) Nuclear localization and increased levels of transcription factor YB-1 in primary human breast cancers are associated with intrinsic MDR1 gene expression. Nat Med 3(4): 447–450 9095180 10.1038/nm0497-447

[bib4] Berquin IM, Pang B, Dziubinski ML, Scott LM, Chen YQ, Nolan GP, Ethier SP (2005) Y-box-binding protein 1 confers EGF independence to human mammary epithelial cells. Oncogene 24(19): 3177–3186 15735691 10.1038/sj.onc.1208504

[bib5] Bonner JA, Harari PM, Giralt J, Azarnia N, Shin DM, Cohen RB, Jones CU, Sur R, Raben D, Jassem J, Ove R, Kies MS, Baselga J, Youssoufian H, Amellal N, Rowinsky EK, Ang KK (2006) Radiotherapy plus cetuximab for squamous-cell carcinoma of the head and neck. N Engl J Med 354(6): 567–578 16467544 10.1056/NEJMoa053422

[bib6] Bozec A, Gros FX, Penault-Llorca F, Formento P, Cayre A, Dental C, Etienne-Grimaldi MC, Fischel JL, Milano G (2008) Vertical VEGF targeting: A combination of ligand blockade with receptor tyrosine kinase inhibition. Eur J Cancer 44(13): 1922–1930 18691881 10.1016/j.ejca.2008.07.013

[bib7] Bryne M, Koppang HS, Lilleng R, Kjaerheim A (1992) Malignancy grading of the deep invasive margins of oral squamous cell carcinomas has high prognostic value. J Pathol 166(4): 375–381 1517891 10.1002/path.1711660409

[bib8] Chang SS, Califano J (2008) Current status of biomarkers in head and neck cancer. J Surg Oncol 97(8): 640–643 18493942 10.1002/jso.21023

[bib9] Chin D, Boyle GM, Williams RM, Ferguson K, Pandeya N, Pedley J, Campbell CM, Theile DR, Parsons PG, Coman WB (2005) Novel markers for poor prognosis in head and neck cancer. Int J Cancer 113(5): 789–797 15499618 10.1002/ijc.20608

[bib10] Chung CH, Parker JS, Ely K, Carter J, Yi Y, Murphy BA, Ang KK, El-Naggar AK, Zanation AM, Cmelak AJ, Levy S, Slebos RJ, Yarbrough WG (2006) Gene expression profiles identify epithelial-to-mesenchymal transition and activation of nuclear factor-kappaB signaling as characteristics of a high-risk head and neck squamous cell carcinoma. Cancer Res 66(16): 8210–8218 16912200 10.1158/0008-5472.CAN-06-1213

[bib11] Coles LS, Lambrusco L, Burrows J, Hunter J, Diamond P, Bert AG, Vadas MA, Goodall GJ (2005) Phosphorylation of cold shock domain/Y-box proteins by ERK2 and GSK3beta and repression of the human VEGF promoter. FEBS Lett 579(24): 5372–5378 16198352 10.1016/j.febslet.2005.08.075

[bib12] Conley BA (2006) Treatment of advanced head and neck cancer: what lessons have we learned? J Clin Oncol 24(7): 1023–1025 16505419 10.1200/JCO.2005.05.0682

[bib13] Das S, Chattopadhyay R, Bhakat KK, Boldogh I, Kohno K, Prasad R, Wilson SH, Hazra TK (2007) Stimulation of NEIL2-mediated oxidized base excision repair via YB-1 interaction during oxidative stress. J Biol Chem 282(39): 28474–28484 17686777 10.1074/jbc.M704672200 PMC2679419

[bib14] Dhillon J, Astanehe A, Lee C, Fotovati A, Hu K, Dunn SE (2010) The expression of activated Y-box binding protein-1 serine 102 mediates trastuzumab resistance in breast cancer cells by increasing CD44(+) cells. Oncogene 29(47): 6294–6300 20802512 10.1038/onc.2010.365

[bib15] Evdokimova V, Ruzanov P, Imataka H, Raught B, Svitkin Y, Ovchinnikov LP, Sonenberg N (2001) The major mRNA-associated protein YB-1 is a potent 5′ cap-dependent mRNA stabilizer. EMBO J 20(19): 5491–5502 11574481 10.1093/emboj/20.19.5491 PMC125650

[bib16] Evdokimova V, Tognon C, Ng T, Ruzanov P, Melnyk N, Fink D, Sorokin A, Ovchinnikov LP, Davicioni E, Triche TJ, Sorensen PH (2009) Translational activation of snail1 and other developmentally regulated transcription factors by YB-1 promotes an epithelial-mesenchymal transition. Cancer Cell 15(5): 402–415 19411069 10.1016/j.ccr.2009.03.017

[bib17] Finkbeiner MR, Astanehe A, To K, Fotovati A, Davies AH, Zhao Y, Jiang H, Stratford AL, Shadeo A, Boccaccio C, Comoglio P, Mertens PR, Eirew P, Raouf A, Eaves CJ, Dunn SE (2009) Profiling YB-1 target genes uncovers a new mechanism for MET receptor regulation in normal and malignant human mammary cells. Oncogene 28(11): 1421–1431 19151767 10.1038/onc.2008.485

[bib18] Forastiere A, Koch W, Trotti A, Sidransky D (2001) Head and neck cancer. N Engl J Med 345(26): 1890–1900 11756581 10.1056/NEJMra001375

[bib19] Gaudreault I, Guay D, Lebel M (2004) YB-1 promotes strand separation *in vitro* of duplex DNA containing either mispaired bases or cisplatin modifications, exhibits endonucleolytic activities and binds several DNA repair proteins. Nucleic Acids Res 32(1): 316–327 14718551 10.1093/nar/gkh170 PMC373280

[bib20] Gimenez-Bonafe P, Fedoruk MN, Whitmore TG, Akbari M, Ralph JL, Ettinger S, Gleave ME, Nelson CC (2004) YB-1 is upregulated during prostate cancer tumor progression and increases P-glycoprotein activity. Prostate 59(3): 337–349 15042610 10.1002/pros.20023

[bib21] Ginos MA, Page GP, Michalowicz BS, Patel KJ, Volker SE, Pambuccian SE, Ondrey FG, Adams GL, Gaffney PM (2004) Identification of a gene expression signature associated with recurrent disease in squamous cell carcinoma of the head and neck. Cancer Res 64(1): 55–63 14729608 10.1158/0008-5472.can-03-2144

[bib22] Gluz O, Mengele K, Schmitt M, Kates R, Diallo-Danebrock R, Neff F, Royer HD, Eckstein N, Mohrmann S, Ting E, Kiechle M, Poremba C, Nitz U, Harbeck N (2009) Y-box-binding protein YB-1 identifies high-risk patients with primary breast cancer benefiting from rapidly cycled tandem high-dose adjuvant chemotherapy. J Clin Oncol 27(36): 6144–6145 19901122 10.1200/JCO.2008.19.6261

[bib23] Habibi G, Leung S, Law JH, Gelmon K, Masoudi H, Turbin D, Pollak M, Nielsen TO, Huntsman D, Dunn SE (2008) Redefining prognostic factors for breast cancer: YB-1 is a stronger predictor of relapse and disease-specific survival than estrogen receptor or HER-2 across all tumor subtypes. Breast Cancer Res 10(5): R86 18925950 10.1186/bcr2156 PMC2614522

[bib24] Haddad RI, Shin DM (2008) Recent advances in head and neck cancer. N Engl J Med 359(11): 1143–1154 18784104 10.1056/NEJMra0707975

[bib25] Hall SF, Groome PA, Irish J, O′Sullivan B (2009) TNM-based stage groupings in head and neck cancer: application in cancer of the hypopharynx. Head Neck 31(1): 1–8 19031408 10.1002/hed.20917

[bib26] Holzmuller R, Mantwill K, Haczek C, Rognoni E, Anton M, Kasajima A, Weichert W, Treue D, Lage H, Schuster T, Schlegel J, Gansbacher B, Holm PS (2011) YB-1 dependent virotherapy in combination with temozolomide as a multimodal therapy approach to eradicate malignant glioma. Int J Cancer 129(5): 1265–1276 21710499 10.1002/ijc.25783

[bib27] Huang CH, Yang WH, Chang SY, Tai SK, Tzeng CH, Kao JY, Wu KJ, Yang MH (2009) Regulation of membrane-type 4 matrix metalloproteinase by SLUG contributes to hypoxia-mediated metastasis. Neoplasia (New York, NY 11(12): 1371–1382 10.1593/neo.91326 PMC2794518 20019845

[bib28] Hundsdorfer B, Zeilhofer HF, Bock KP, Dettmar P, Schmitt M, Kolk A, Pautke C, Horch HH (2005) Tumour-associated urokinase-type plasminogen activator (uPA) and its inhibitor PAI-1 in normal and neoplastic tissues of patients with squamous cell cancer of the oral cavity - clinical relevance and prognostic value. J Craniomaxillofac Surg 33(3): 191–196 15878520 10.1016/j.jcms.2004.12.005

[bib29] Janz M, Harbeck N, Dettmar P, Berger U, Schmidt A, Jurchott K, Schmitt M, Royer HD (2002) Y-box factor YB-1 predicts drug resistance and patient outcome in breast cancer independent of clinically relevant tumor biologic factors HER2, uPA and PAI-1. Inte J Cancer 97(3): 278–282 10.1002/ijc.1610 11774277

[bib30] Jethwa P, Naqvi M, Hardy RG, Hotchin NA, Roberts S, Spychal R, Tselepis C (2008) Overexpression of Slug is associated with malignant progression of esophageal adenocarcinoma. World J Gastroenterol 14(7): 1044–1052 18286686 10.3748/wjg.14.1044 PMC2689407

[bib31] Jurchott K, Bergmann S, Stein U, Walther W, Janz M, Manni I, Piaggio G, Fietze E, Dietel M, Royer HD (2003) YB-1 as a cell cycle-regulated transcription factor facilitating cyclin A and cyclin B1 gene expression. J Biol Chem 278(30): 27988–27996 12695516 10.1074/jbc.M212966200

[bib32] Kamura T, Yahata H, Amada S, Ogawa S, Sonoda T, Kobayashi H, Mitsumoto M, Kohno K, Kuwano M, Nakano H (1999) Is nuclear expression of Y box-binding protein-1 a new prognostic factor in ovarian serous adenocarcinoma? Cancer 85(11): 2450–2454 10357417 10.1002/(sici)1097-0142(19990601)85:11<2450::aid-cncr21>3.0.co;2-u

[bib33] Kashihara M, Azuma K, Kawahara A, Basaki Y, Hattori S, Yanagawa T, Terazaki Y, Takamori S, Shirouzu K, Aizawa H, Nakano K, Kage M, Kuwano M, Ono M (2009) Nuclear Y-box binding protein-1, a predictive marker of prognosis, is correlated with expression of HER2/ErbB2 and HER3/ErbB3 in non-small cell lung cancer. J Thorac Oncol 4(9): 1066–1074 19648825 10.1097/JTO.0b013e3181ae2828

[bib34] Kohno K, Izumi H, Uchiumi T, Ashizuka M, Kuwano M (2003) The pleiotropic functions of the Y-box-binding protein, YB-1. Bioessays 25(7): 691–698 12815724 10.1002/bies.10300

[bib35] Kuwano M, Oda Y, Izumi H, Yang SJ, Uchiumi T, Iwamoto Y, Toi M, Fujii T, Yamana H, Kinoshita H, Kamura T, Tsuneyoshi M, Yasumoto K, Kohno K (2004) The role of nuclear Y-box binding protein 1 as a global marker in drug resistance. Mol Cancer Ther 3(11): 1485–1492 15542787

[bib36] Kuwano M, Uchiumi T, Hayakawa H, Ono M, Wada M, Izumi H, Kohno K (2003) The basic and clinical implications of ABC transporters, Y-box-binding protein-1 (YB-1) and angiogenesis-related factors in human malignancies. Cancer Sci 94(1): 9–14 12708467 10.1111/j.1349-7006.2003.tb01344.x PMC11160199

[bib37] Langer R, Von Rahden BH, Nahrig J, Von Weyhern C, Reiter R, Feith M, Stein HJ, Siewert JR, Hofler H, Sarbia M (2006) Prognostic significance of expression patterns of c-erbB-2, p53, p16INK4A, p27KIP1, cyclin D1 and epidermal growth factor receptor in oesophageal adenocarcinoma: a tissue microarray study. J Clin Pathol 59(6): 631–634 16731604 10.1136/jcp.2005.034298 PMC1860401

[bib38] Lee C, Dhillon J, Wang MY, Gao Y, Hu K, Park E, Astanehe A, Hung MC, Eirew P, Eaves CJ, Dunn SE (2008) Targeting YB-1 in HER-2 overexpressing breast cancer cells induces apoptosis via the mTOR/STAT3 pathway and suppresses tumor growth in mice. Cancer Res 68(21): 8661–8666 18974106 10.1158/0008-5472.CAN-08-1082

[bib39] Li QQ, Xu JD, Wang WJ, Cao XX, Chen Q, Tang F, Chen ZQ, Liu XP, Xu ZD (2009) Twist1-mediated adriamycin-induced epithelial-mesenchymal transition relates to multidrug resistance and invasive potential in breast cancer cells. Clin Cancer Res 15(8): 2657–2665 19336515 10.1158/1078-0432.CCR-08-2372

[bib40] Ludwig JA, Weinstein JN (2005) Biomarkers in cancer staging, prognosis and treatment selection. Nat Rev Cancer 5(11): 845–856 16239904 10.1038/nrc1739

[bib41] Masuda M, Wakasaki T, Suzui M, Toh S, Joe AK, Weinstein IB (2010) Stat3 orchestrates tumor development and progression: the Achilles′ heel of head and neck cancers? Curr Cancer Drug Targets 10(1): 117–126 20088788 10.2174/156800910790980197

[bib42] Mertens PR, Harendza S, Pollock AS, Lovett DH (1997) Glomerular mesangial cell-specific transactivation of matrix metalloproteinase 2 transcription is mediated by YB-1. J Biol Chem 272(36): 22905–22912 9278454 10.1074/jbc.272.36.22905

[bib43] Mucke T, Wagenpfeil S, Kesting MR, Holzle F, Wolff KD (2009) Recurrence interval affects survival after local relapse of oral cancer. Oral Oncol 45(8): 687–691 19095488 10.1016/j.oraloncology.2008.10.011

[bib44] Oda Y, Ohishi Y, Saito T, Hinoshita E, Uchiumi T, Kinukawa N, Iwamoto Y, Kohno K, Kuwano M, Tsuneyoshi M (2003) Nuclear expression of Y-box-binding protein-1 correlates with P-glycoprotein and topoisomerase II alpha expression, and with poor prognosis in synovial sarcoma. J Pathol 199(2): 251–258 12533839 10.1002/path.1282

[bib45] Oda Y, Sakamoto A, Shinohara N, Ohga T, Uchiumi T, Kohno K, Tsuneyoshi M, Kuwano M, Iwamoto Y (1998) Nuclear expression of YB-1 protein correlates with P-glycoprotein expression in human osteosarcoma. Clin Cancer Res 4(9): 2273–2277 9748149

[bib46] Pham CG, Bubici C, Zazzeroni F, Knabb JR, Papa S, Kuntzen C, Franzoso G (2007) Upregulation of Twist-1 by NF-kappaB blocks cytotoxicity induced by chemotherapeutic drugs. Mol Cell Biol 27(11): 3920–3935 17403902 10.1128/MCB.01219-06 PMC1900008

[bib47] Prince ME, Sivanandan R, Kaczorowski A, Wolf GT, Kaplan MJ, Dalerba P, Weissman IL, Clarke MF, Ailles LE (2007) Identification of a subpopulation of cells with cancer stem cell properties in head and neck squamous cell carcinoma. Proc Natl Acad Sci USA 104(3): 973–978 17210912 10.1073/pnas.0610117104 PMC1783424

[bib48] Robbins KT, Shaha AR, Medina JE, Califano JA, Wolf GT, Ferlito A, Som PM, Day TA (2008) Consensus statement on the classification and terminology of neck dissection. Arch Otolaryngol Head Neck Surg 134(5): 536–538 18490577 10.1001/archotol.134.5.536

[bib49] Rogers SN, Brown JS, Woolgar JA, Lowe D, Magennis P, Shaw RJ, Sutton D, Errington D, Vaughan D (2009) Survival following primary surgery for oral cancer. Oral Oncol 45(3): 201–211 18674959 10.1016/j.oraloncology.2008.05.008

[bib50] Schittek B, Psenner K, Sauer B, Meier F, Iftner T, Garbe C (2007) The increased expression of Y box-binding protein 1 in melanoma stimulates proliferation and tumor invasion, antagonizes apoptosis and enhances chemoresistance. Int J Cancer 120(10): 2110–2118 17266041 10.1002/ijc.22512

[bib51] Shah NG, Trivedi TI, Tankshali RA, Goswami JA, Jetly DH, Kobawala TP, Shukla SN, Shah PM, Verma RJ (2006) Stat3 expression in oral squamous cell carcinoma: association with clinicopathological parameters and survival. Int J Biol Markers 21(3): 175–183 17013800 10.1177/172460080602100307

[bib52] Shah NG, Trivedi TI, Tankshali RA, Goswami JV, Jetly DH, Shukla SN, Shah PM, Verma RJ (2009) Prognostic significance of molecular markers in oral squamous cell carcinoma: a multivariate analysis. Head Neck 31(12): 1544–1556 19424974 10.1002/hed.21126

[bib53] Sherry MM, Reeves A, Wu JK, Cochran BH (2009) STAT3 is required for proliferation and maintenance of multipotency in glioblastoma stem cells. Stem cells (Dayton, Ohio) 27(10): 2383–2392 10.1002/stem.185 PMC4391626 19658181

[bib54] Shibahara K, Sugio K, Osaki T, Uchiumi T, Maehara Y, Kohno K, Yasumoto K, Sugimachi K, Kuwano M (2001) Nuclear expression of the Y-box binding protein, YB-1, as a novel marker of disease progression in non-small cell lung cancer. Clin Cancer Res 7(10): 3151–3155 11595709

[bib55] Shiota M, Izumi H, Tanimoto A, Takahashi M, Miyamoto N, Kashiwagi E, Kidani A, Hirano G, Masubuchi D, Fukunaka Y, Yasuniwa Y, Naito S, Nishizawa S, Sasaguri Y, Kohno K (2009) Programmed cell death protein 4 down-regulates Y-box binding protein-1 expression via a direct interaction with Twist1 to suppress cancer cell growth. Cancer Res 69(7): 3148–3156 19318582 10.1158/0008-5472.CAN-08-2334

[bib56] Stickeler E, Fraser SD, Honig A, Chen AL, Berget SM, Cooper TA (2001) The RNA binding protein YB-1 binds A/C-rich exon enhancers and stimulates splicing of the CD44 alternative exon v4. EMBO J 20(14): 3821–3830 11447123 10.1093/emboj/20.14.3821 PMC125550

[bib57] Stratford AL, Habibi G, Astanehe A, Jiang H, Hu K, Park E, Shadeo A, Buys TP, Lam W, Pugh T, Marra M, Nielsen TO, Klinge U, Mertens PR, Aparicio S, Dunn SE (2007) Epidermal growth factor receptor (EGFR) is transcriptionally induced by the Y-box binding protein-1 (YB-1) and can be inhibited with Iressa in basal-like breast cancer, providing a potential target for therapy. Breast Cancer Res 9(5): R61 17875215 10.1186/bcr1767 PMC2242657

[bib58] Sturgis EM, Wei Q, Spitz MR (2004) Descriptive epidemiology and risk factors for head and neck cancer. Semin Oncol 31(6): 726–733 15599850 10.1053/j.seminoncol.2004.09.013

[bib59] Sutherland BW, Kucab J, Wu J, Lee C, Cheang MC, Yorida E, Turbin D, Dedhar S, Nelson C, Pollak M, Leighton Grimes H, Miller K, Badve S, Huntsman D, Blake-Gilks C, Chen M, Pallen CJ, Dunn SE (2005) Akt phosphorylates the Y-box binding protein 1 at Ser102 located in the cold shock domain and affects the anchorage-independent growth of breast cancer cells. Oncogene 24(26): 4281–4292 15806160 10.1038/sj.onc.1208590

[bib60] Tay WL, Yip GW, Tan PH, Matsumoto K, Yeo R, Ng TP, Kumar SD, Tsujimoto M, Bay BH (2009) Y-Box-binding protein-1 is a promising predictive marker of radioresistance and chemoradioresistance in nasopharyngeal cancer. Mod Pathol 22(2): 282–290 18978732 10.1038/modpathol.2008.181

[bib61] Tepper J, Krasna MJ, Niedzwiecki D, Hollis D, Reed CE, Goldberg R, Kiel K, Willett C, Sugarbaker D, Mayer R (2008) Phase III trial of trimodality therapy with cisplatin, fluorouracil, radiotherapy, and surgery compared with surgery alone for esophageal cancer: CALGB 9781. J Clin Oncol 26(7): 1086–1092 18309943 10.1200/JCO.2007.12.9593 PMC5126644

[bib62] To K, Fotovati A, Reipas KM, Law JH, Hu K, Wang J, Astanehe A, Davies AH, Lee L, Stratford AL, Raouf A, Johnson P, Berquin IM, Royer HD, Eaves CJ, Dunn SE (2010) Y-box binding protein-1 induces the expression of CD44 and CD49f leading to enhanced self-renewal, mammosphere growth, and drug resistance. Cancer Res 70(7): 2840–2851 20332234 10.1158/0008-5472.CAN-09-3155 PMC2848879

[bib63] Wu J, Stratford AL, Astanehe A, Dunn SE (2007) YB-1 is a transcription/translation factor that orchestrates the oncogenome by hardwiring signal transduction to gene expression. Transl Oncogen 2007: 249–265 PMC3634714 23641145

[bib64] Xie F, Li K, Ouyang X (2009) Twist, an independent prognostic marker for predicting distant metastasis and survival rates of esophageal squamous cell carcinoma patients. Clin Exp Metastasis 26(8): 1025–1032 19816777 10.1007/s10585-009-9292-5

[bib65] Xu W, Zhou L, Qin R, Tang H, Shen H (2009) Nuclear expression of YB-1 in diffuse large B-cell lymphoma: correlation with disease activity and patient outcome. Eur J Haematol 83(4): 313–319 19500133 10.1111/j.1600-0609.2009.01285.x

[bib66] Yang J, Mani SA, Donaher JL, Ramaswamy S, Itzykson RA, Come C, Savagner P, Gitelman I, Richardson A, Weinberg RA (2004) Twist, a master regulator of morphogenesis, plays an essential role in tumor metastasis. Cell 117(7): 927–939 15210113 10.1016/j.cell.2004.06.006

